# Implanted subcutaneous versus intraperitoneal bioscaffold seeded with hepatocyte-like cells: functional evaluation

**DOI:** 10.1186/s13287-021-02531-2

**Published:** 2021-08-06

**Authors:** Amal Elham Fares, Hala Gabr, Asmaa Mohammed ShamsEldeen, Haithem A. M. Farghali, Mazen Mohamed Salem Mohamed Rizk, Bassem Essam Mahmoud, Ahmed Bakr Ahmed Tammam, Ayman Magdy Ahmed Mahmoud, Alaa Abdulfattah Mahmoud Suliman, Mohamed Abdelhamid Ali Ayyad, Sahar Hassan Ahmed, Rokia Mohamad Hassan

**Affiliations:** 1 Histology Department, Faculty of Medicine, Cairo University, Giza, Egypt; 2 Clinical Pathology Department, Faculty of Medicine, Cairo University, Giza, Egypt; 3 Physiology Department, Faculty of Medicine, Cairo University, Giza, Egypt; 4 Surgery, Anesthesiology and Radiology Department, Faculty of Veterinary Medicine, Cairo University, Giza, Egypt; 5 Siparadigm Diagnostics, Montville, NJ USA; 6 Al Abbasya Hospital for Mental Health, Cairo, Egypt; 7 Anesthesia Department, Eldemerdash University Hospital, Cairo, Egypt; 8 Interventional and Diagnostic Radiology Department, Faculty of Medicine, Cairo University, Giza, Egypt; 9 National Cancer Institute, Cairo University, Giza, Egypt; 10 Faculty of Medicine, Cairo University, Giza, Egypt; 11 Medical Laboratory Technology Department, Faculty of Applied Health Science Technology, Misr University for Science and Technology, Giza, Egypt

**Keywords:** Hemophilia A, Bioscaffold, Stem cells, Factor VIII

## Abstract

**Background and objectives:**

The X-linked bleeding disorder, hemophilia A, is caused by defective production of factor VIII (FVIII). Hemophilic patients require regular FVIII infusions. Recombinant factor replacement poses the safest line of therapy. However, its main drawbacks are high expenses and the higher liability for formation of inhibitors. Recent studies confirmed the ability of bone marrow-derived stem cells to secrete FVIII. This study aims to generate bioscaffold from decellularized liver and subsequently seed it with trans-differentiated human stem cells into hepatic-like cells. This scaffold can then be implanted intraperitoneally or subcutaneously to provide FVIII.

**Methods:**

After generation of the bioscaffold, seeding of discoid scaffolds with trans-differentiated human hepatocyte-like cells was performed. Then, the generated organoid was implanted into peritoneal cavity or subcutaneous tissue of experimental rats.

**Results:**

Serum human FVIII was significantly increased in rats subjected to subcutaneous implantation compared intraperitoneal implantation. Immunostaining for detecting Cytokeratin 19 and human anti-globulin confirmed the presence of mature human hepatocytes that were significantly increased in subcutaneous implanted scaffold compared to the intraperitoneal one.

**Conclusion:**

Implantation of decellularized bioscaffold seeded with trans-differentiated stem cells in rats was successful to establish production of FVIII. Subcutaneous implantation showed higher FVIII levels than intraperitoneal implantation.

## Introduction

Hemophilia is an x-linked inherited disease caused by deficiency of clotting factors VIII (Hemophilia A) or IX (Hemophilia B). Hemophilia is associated with recurrent and spontaneous bleeding, mainly internal bleedings into joints or muscles [1]. Hemophilia A occurs in 1 out of 10,000 male births, whereas hemophilia B occurs in 1 out of 30,000 male births. The prevalence of hemophilia A changes with the reporting country, ranging from 5.4 to 14.5 cases per 100,000 male individuals [2].

Management of Hemophilia A is directed mainly toward symptomatic prevention of bleeding through replacement of defective factor. Several clotting factor VIII concentrates are available for managing hemophilia A. Biotechnology is able to develop recombinant factor concentrates for replacement therapy [3].

However, the disadvantages of replacement therapy are its high cost, life-long dependence as well as antibody production [4]. The most serious complication of replacement therapy in hemophilia is antibody production [5]. These antibodies are polyclonal high-affinity immunoglobulin G directed against the factor VIII protein leading to an increase in the management cost, morbidity and mortality [6].

Increasing circulating level of factor VIII by 1–5% could substantially reduce bleeding tending. Being the source of plasma factor VIII, liver cells became the main hope for treating hemophilia A [4]. Moreover, to find a feasible long-life line of treatment such as liver cell based therapy emphasizes a major concern. Recently, Rossi and colleagues have reported improvement viability and functions of transplanted hepatocytes seeded onto naturally developed bioscaffold by decellularization process [7].

The formed bioscaffold is composed of hepatic-extracellular matrix (ECM) such as collagen, elastin and proteoglycans, and no remaining functioning cells, but still rich in factors that could promote cellular interaction, growth and survival [8]. Thus, seeding the decellularized liver bioscaffold with stem cells can enhance their growth, therapeutic efficacy and differentiation into functioning cells [7]

Mesenchymal stem cells (MSCs) represent a feasible cell source for cell therapy for their accessibility, expansion potential, high regenerative capacity, and safety in a large number of clinical trials [9]. Previous studies revealed that MSC-derived from different sources (both mice and humans) can be differentiated into hepatocytes, using various protocols and techniques in vitro [10].

Notably, this study was designed to evaluate the function and histology of decellularized liver bioscaffold seeded with MSC-derived hepatocyte-like cells implanted in rats and compare between subcutaneous and intraperitoneal routes, finally to verify the ability of both scaffolds to survive, function and secrete functional proteins.

## Materials and methods

### Generation of bioscaffold

Surgical procedure: Animals were euthanized, then a longitudinal anterior abdominal incision was made. A 5-kg piglet liver was removed surgically following intraoperative ligation of the hepatic artery and splenic vein to avoid leakage of the detergents used in decellularization process. The liver was dissected with the capsule intact.

### Decellularization process

The liver was dissected and the portal vein was cannulated and connected to pump that perfused the liver with sterile distilled water. 15 L of distilled water was injected in the liver until all blood inside the liver was washed. Subsequently, 6 L of detergent were injected (1% SDS + 0.1% ammonium hydroxide, Sigma Aldrich, St. Louis, USA) to kill the hepatocytes and the endothelial cells. Decellularized liver bioscaffolds were detected by being transparent then confirmed laboratory by the absence of positive staining for hematoxylin [11] of liver sample.

### Mononuclear cell separation from human bone marrow samples

The experiments involving humans were carried out in accordance with The Code of Ethics of the World Medical Association (Human Declaration of Helsinki). After institutional ethical committee approval and taking informed consent from the participants, three-milliliter of bone marrow was obtained by aspiration from the posterior iliac spine. The mononuclear cells were obtained from fresh human bone marrow samples after using Ficoll–Hypaque density gradient (density, 1.077; Biochrom KG, Berlin) [12]. Cell pellets were suspended in 1 mL serum-free DMEM media and counted using a hemocytometer. Counting and viability was assessed by the vital stain trypan blue (0.4%) exclusive dye (Sigma).

### Mesenchymal stem cell (MSCs) separation and identification

**Fig. 1:**
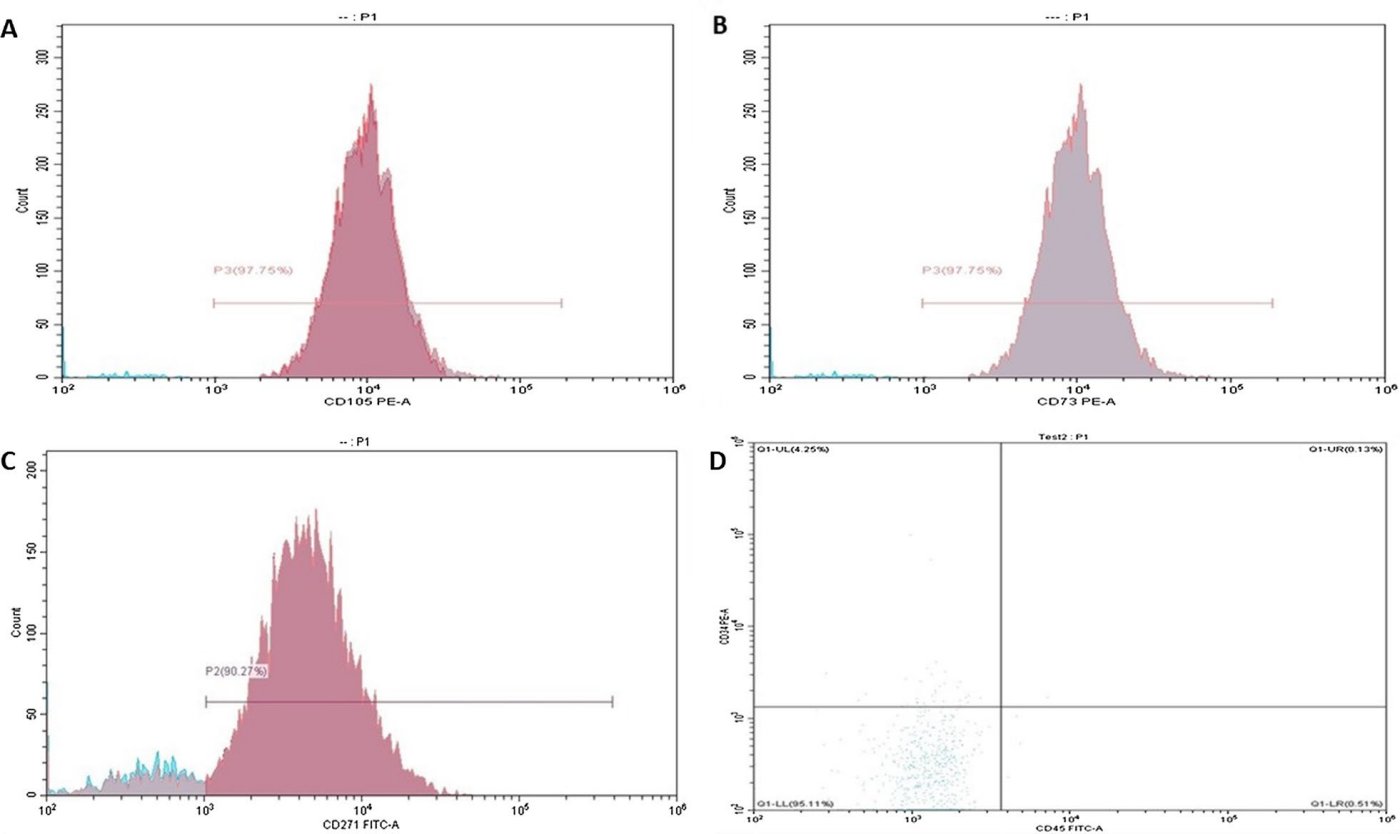
**a**–**d** Immunophenotyping of the isolated human MSCs. Isolated human-MSCs were characterized by morphology and Fluorescence-activated Cell Sorting (FACS) by assessment positivity of CD 105^+^, CD 73^+^, and CD 271^+^ and negativity of CD 45^+^ and CD 34^+^ specific to MSCs

After isolation, primary culture and subculture of MSCs were done according to Soleimani and Nadir technique [13]. Flasks used were incubated in a horizontal position in humidified incubator at 37 °C and 5% CO_2_. The first change of media was accomplished at the third to the fifth day to remove non adherent cells, and then the media was changed twice weekly until reaching 70–90% confluency which was assessed by the inverted microscope. The adherent cells were harvested by trypsinization according to Bieback method [14]. After trypsnization, cells were examined under the microscope, counted and the viability was assessed using the trypan blue dye. The cells released from primary culture were divided into two parts, one used for MSCs identification using flow cytometry and the other part for subculture and induction of hepatocyte differentiation. Flow cytometry was done and results were declared as percentage of cells expressing CD 105^+^, CD 73^+^ and CD 271^+^ and negative for CD 45^+^ and CD 34^+^ in the gated population of mononuclear cells (Fig. 1).

### Induction of hepatocyte differentiation

Induction of hepatocyte differentiation was done according to Li method [15]. At this step, 14 samples were incubated with hepatocyte growth factor (HGF) 20 ng/mL together with fibroblast growth factor (FGF) 10 ng/mL for induction of differentiation, whereas another six samples were used as a control.

### Evaluation of hepatocyte differentiation

Evaluation of hepatocyte differentiation was done using several measures firstly under inverted microscope, and dishes were examined for morphological changes denoting differentiation.

### Gene expression of AFP and albumin was detected using real time: PCR

The total RNA was extracted from hepatic-like cells according to the manufacture instructions. In brief, the first denaturation step was done by incubating reaction tubes for 10 min at 94 °C, then followed by 40 cycles of 1 min at 94 °C (denaturation). For AFP and albumin, 1 min was applied for 58 °C and 56 °C for β-actin (annealing) and 1 min at 72 °C (extension) and the final elongation step was at 72 °C for 7 min. The primer sequence for AFP is sense; ACCATGAAGTGGGTGGAATC, and antisense; TGGTAGCCAGGTCAGCTAAA, for albumin is sense; GCATCCTGATTACTCTGTCG, and antisense; GTTCACCAAGGATTCTGTGC, and for β-actin is sense; GGGCATGGGTCAGAAGGATT, and antisense; GAGGCGTACAGGGATAGCAC.

On day 14, the media were condensed five times by freeze drying before estimating concentrations of albumin and AFP [16]. Albumin and AFP were also measured in the samples using radioimmunoassay method. AFP was measured using AFP monoclonal antibody (P5B8) a product of ThermoFisher Cat #MA5-14666, and albumin was measured using human serum albumin monoclonal antibody (1E1) a product of TheimoFisher Cat # MIH3001.

### Seeding the formed scaffolds with differentiated MSCs

The differentiated MSCs were resuspended at a concentration of 2 × 10^6^/50 μL/cubic scaffold. Using a 0.5 mL insulin syringe, cells were drawn up then released drop by drop over scaffolds to cover the prepared decellularized tissue.

### Animal experimental design

All animal experiments complied with the ARRIVE guidelines and carried out in accordance with the U.K. Animals (Scientific Procedures) Act, 1986 and associated guidelines, EU Directive 2010/63/EU for animal experiments. This study was conducted on 21 adult male albino rats with average body weight 200 g, purchased from and housed in the Animal House, Faculty of Medicine, Cairo University, and experimental procedures were done in Physiology laboratory, Faculty of medicine according to the guidelines for the care and use of experimental animals of Cairo University and were divided into the following three groups: Group I (control): It included seven rats that were left without any intervention. Group II (subcutaneous group): It included seven rats that were implanted subcutaneously with the generated bioscaffold after differentiation in culture for14 days. Group III (Intraperitoneal group): It included seven rats that were implanted intraperitoneally with the generated bioscaffold after differentiation in culture for 14 days.

#### Laboratory investigation

By the end of the study (after 10 days), blood samples were collected and plasma was separated for detecting level of FVIII using human FVIII ELISA kit (total FVIII antigen) (ab272771). The detection method was colorimetric. Rats of all groups were sacrificed after 10 days. The rats were sacrificed by cervical dislocation. Skin and peritoneal specimens were fixed in 10% formol saline for 24 h. Paraffin blocks were prepared and 5-μm-thick serial sections were subjected to the following studies in the Histology Department, Faculty of Medicine, Cairo University:

#### Histological study

After preparation of paraffin blocks, serial sections from all studied groups were stained with; 1-Hematoxylin and eosin (H&E) 2-Immunohistochemical Study: using the following: a-Alpha-fetoprotein (AFP), (catalog # MBS173005) MyBioSource company, Southern California, San Diego, USA) for early progenitor stem cells. b-Cytokeratin 19, (Mouse Anti-CK19 antibody), purchased from Biological life science Company, USA (Catalog # 381146), for detecting oval cell which is bipotent, can differentiate for hepatocyte and cholangiocyte. 3-Human anti-globulin (HAG), rabbit Anti-Human IgG antibody (ab98568, Cambridge, UK), for detecting of mature human hepatocytes.

#### Morphometric study

Computer-assisted image analysis was performed using Olympus camera connected to Olympus microscope, using interactive measurements menu. The assessment of area percent (%) of AFP, CK19 (in both culture tissue and experimental groups) &HAG was measured using binary mode. The measurements were done within 10 non-overlapping fields of each specimen using × 100 magnification.

#### Statistical methods

Statistical analysis and calculations were performed using Statistical Package for the Social Sciences (SPSS) version 16. The comparison between the different groups was analyzed using ANOVA test, followed by Bonferroni post hoc test to detect which pairs of groups caused the significant difference. *P* values < 0.05 were considered statistically significant.

## Results

### Bioscaffold quality control and characterization

**Fig. 2:**
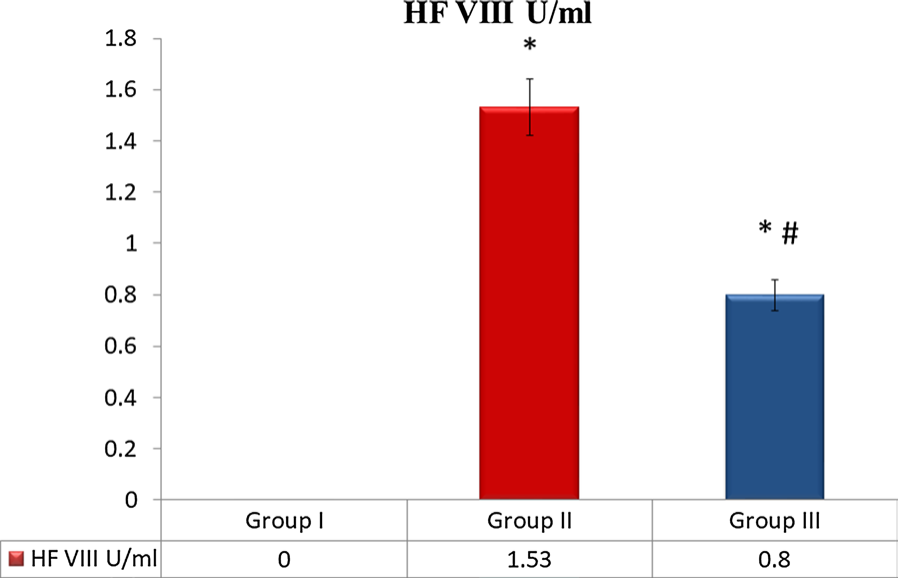
Estimated serum levels of human factor VIII in rats subjected to subcutaneous and intraperitoneal implantation of bioscaffold. Data presented as SD. *Statistically significant compared to the group I (*P* < 0.001). ^#^Statistically significant compared to the group II (*P* < 0.001)

Enhanced production of human factor VIII with implantation of bioscaffolds: Therapeutic potential of implanted bioscaffolds documented production of human factor VIII in group II and III. However, serum level of HF VIII was significantly increased in rats subjected to subcutaneous compared to animals received bioscaffold by intraperitoneal implantation (Fig. 2).

### Cell morphology presenting differentiation of MSCs

**Fig. 3:**
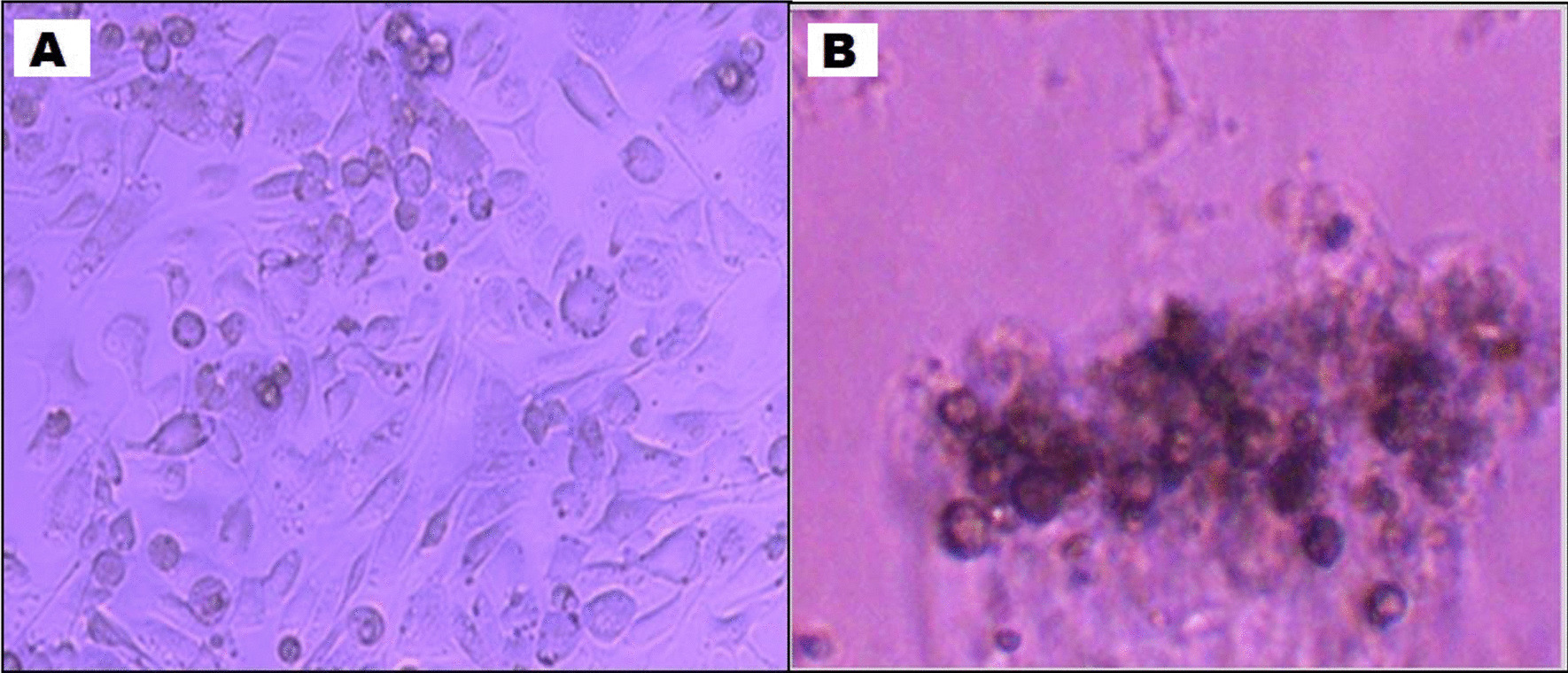
Inverted microscope image of human mesenchymal stem cells showing in **a** Undifferentiation, in **b** Differentiated mesenchymal stem cells into hepatic phenotype cells (× 400). The differentiated stem cells aggregated to form (clusters) cell colonies, and morphological characterized by having large polygonal morphology with round nuclei

Differentiation of human mesenchymal stem cells to hepatic phenotype was confirmed by inverted microscope (Fig. 3).

### Culture results

**Fig. 4:**
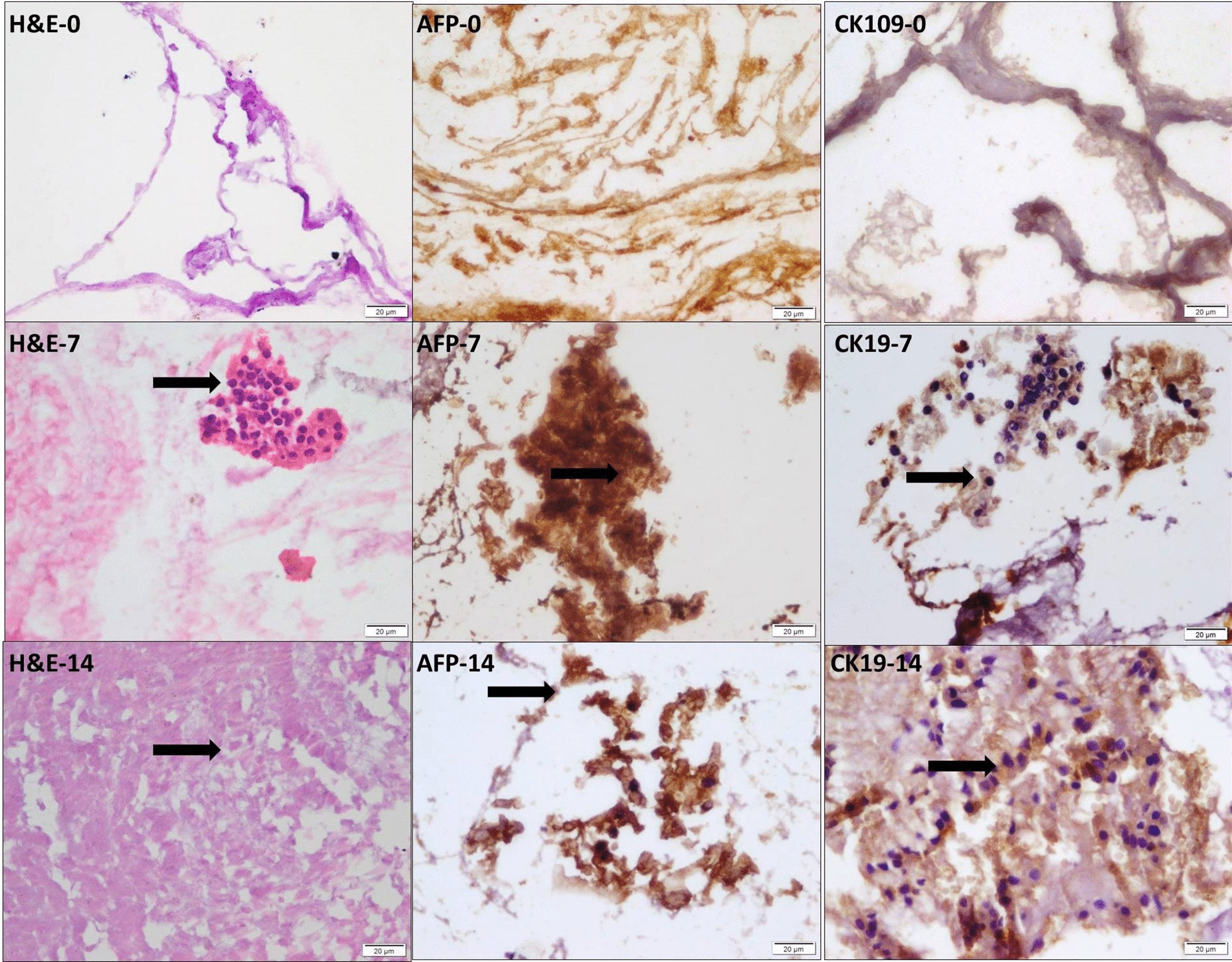
Photomicrographs of cellular differentiation of the culture scaffold at day 0, 7 and 14 using H&E stain, immunohistochemical stain for both AFP and CK19 for detection of differentiated hepatocyte-like cells (arrows) showing at day 0 no detected differentiated cells, at day 7 showing increased number of differentiated cells H&E, AFP & in CK19 and at day 14 the cells increase in H&E and in CK19 while decreased in AFP immunostaining (× 400)

Examinations of cellular differentiation for detection of differentiated hepatocyte-like cells of the culture scaffold was done using H&E and immunohistochemical stains for both alpha-fetoprotein (AFP) and cytokeratin 19 (CK19). Results showed on day 0 no differentiated cells detected, on day 7 increased numbers of differentiated cells and on day 14 the cells increased in H&E and in CK19 while decreased in AFP immunostaining (Fig. 4).

### Estimation of albumin and AFP secretion in culture media

Data confirmed successful induction of differentiation of human bone marrow mesenchymal stem cells (HBMSCs). That was reflected on significant increase in mean value of both albumin (1.06 ± 0.03 µg/mL) and AFP (20.8 ± 0.94 µg/mL) in culture media of differentiated stem cells compared to the mean values of albumin (0.2 ± 0.01 µg/mL) and in AFP (12.93 ± 0.62 µg/mL) estimated in culture media of the control undifferentiated cells.

### Histological results

**Fig. 5:**
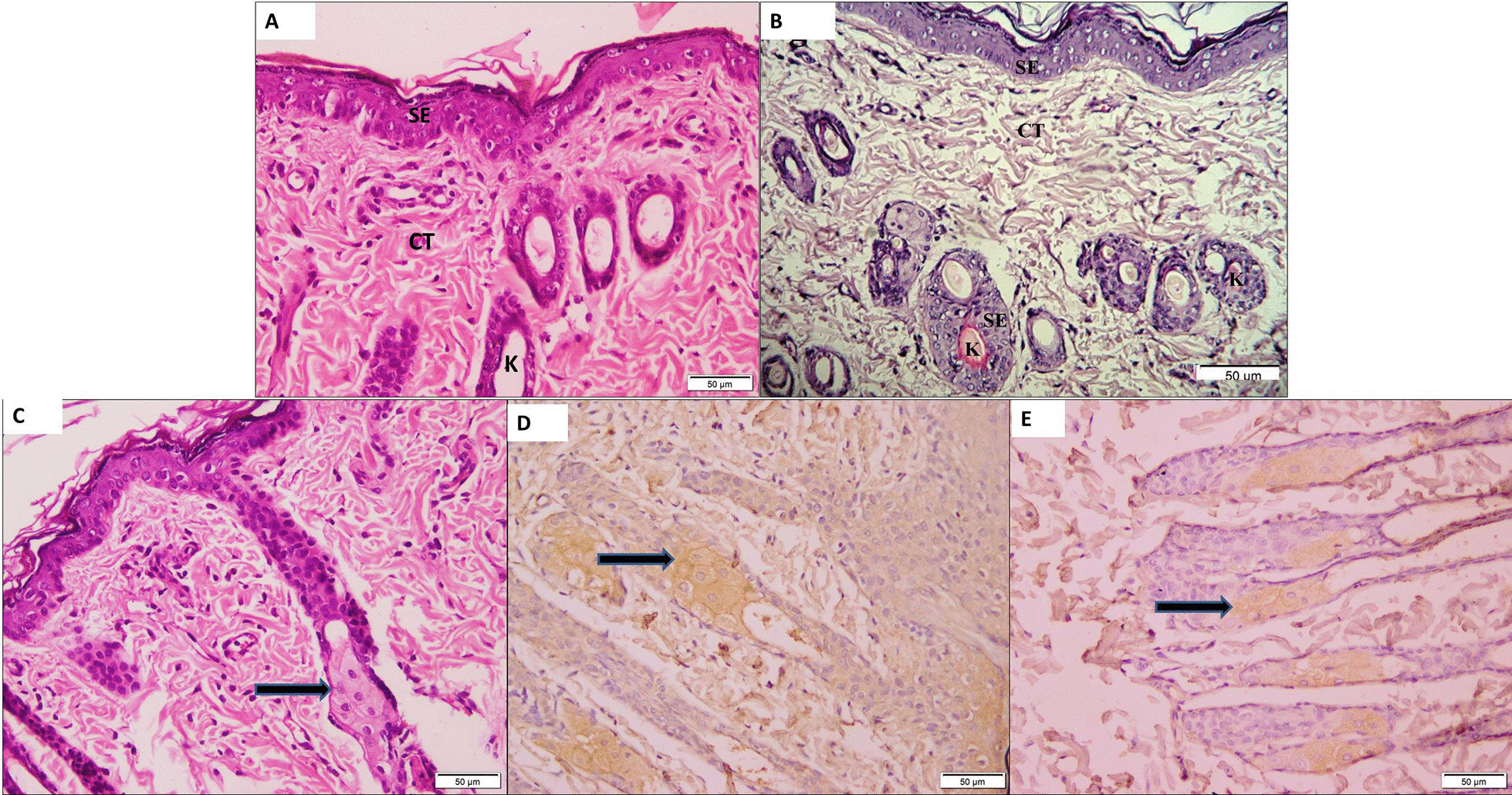
Photomicrographs from Group I (control group): **a** H&E-stained sections showing normal stratified epithelium of epidermal (SE) and dermal (CT) layers with normal hair follicles (K) and thin keratin layer (curved arrow). **b** Immunostained sections showing −ve skin reaction. Photomicrographs from Group II (subcutaneous group): **c** H&E-stained showing hepatocyte-like cells (arrow) at the base of a hair follicle. **d** Stained sections with AFP showing +ve immunostaining cells (arrow) detected at the root of hair follicles. **e** Stained sections with CK19 showing +ve immunostaining cells (arrow) detected at the shaft of hair follicles (× 200)

**Fig. 6:**
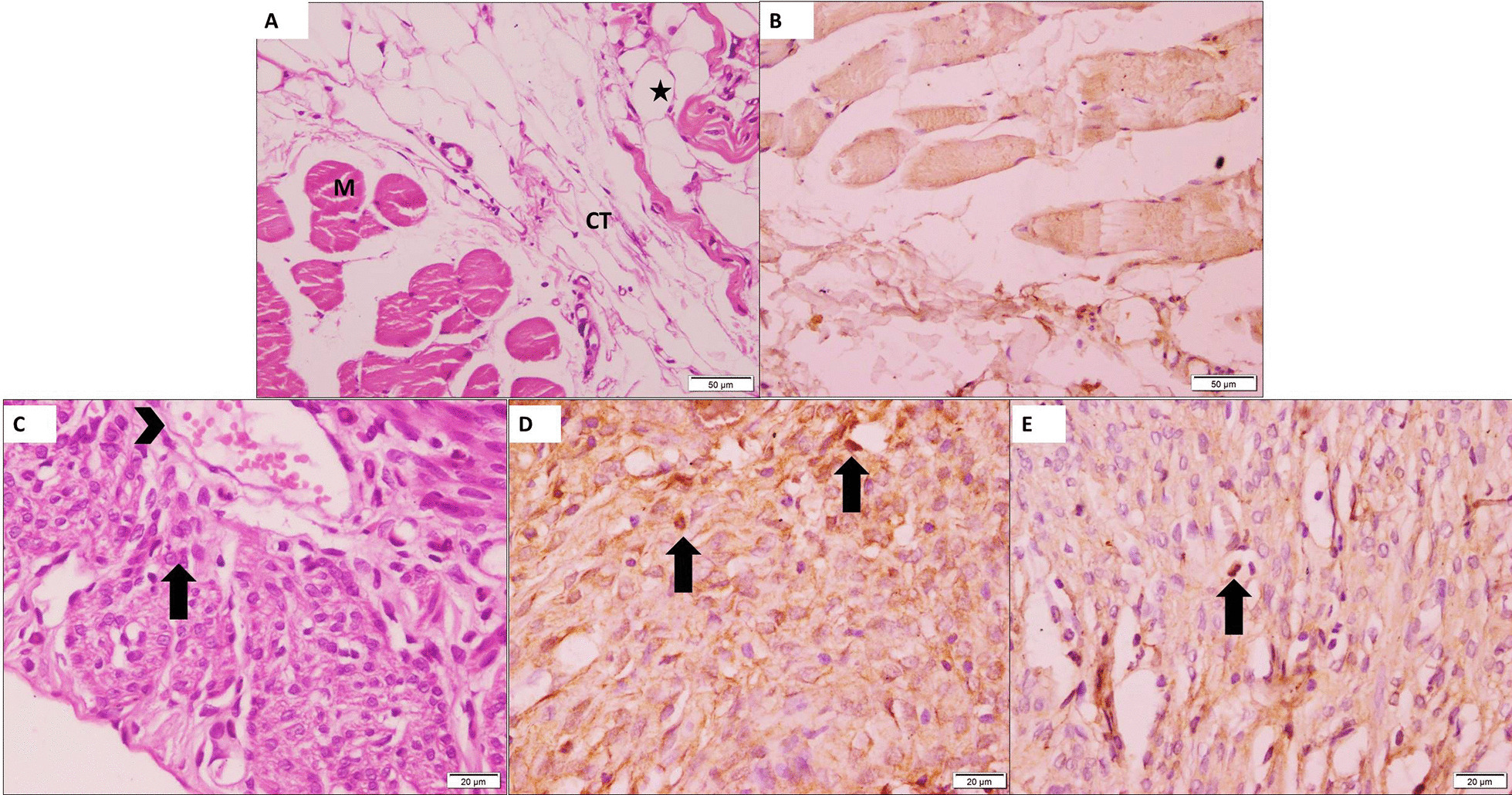
Photomicrographs from Group I (control group): **a** H&E-stained sections showing normal intraperitoneal tissue with some adipose cells (star) and some muscle fibers (M) within loose CT layer (CT). **b** Immunostained sections showing −ve intraperitoneal reactions. Photomicrographs from Group III (intraperitoneal group) showing **c** H&E-stained sections with hepatocyte-like cells (arrow) at the scaffold. Note, the dilated blood vessel (arrowhead) D: Stained sections with AFP showing +ve immunostaining cells (arrows) detected at the scaffold. **e** Stained sections with CK19 showing +ve immunostaining cells (arrow) detected at the scaffold (× 200)

**Fig. 7:**
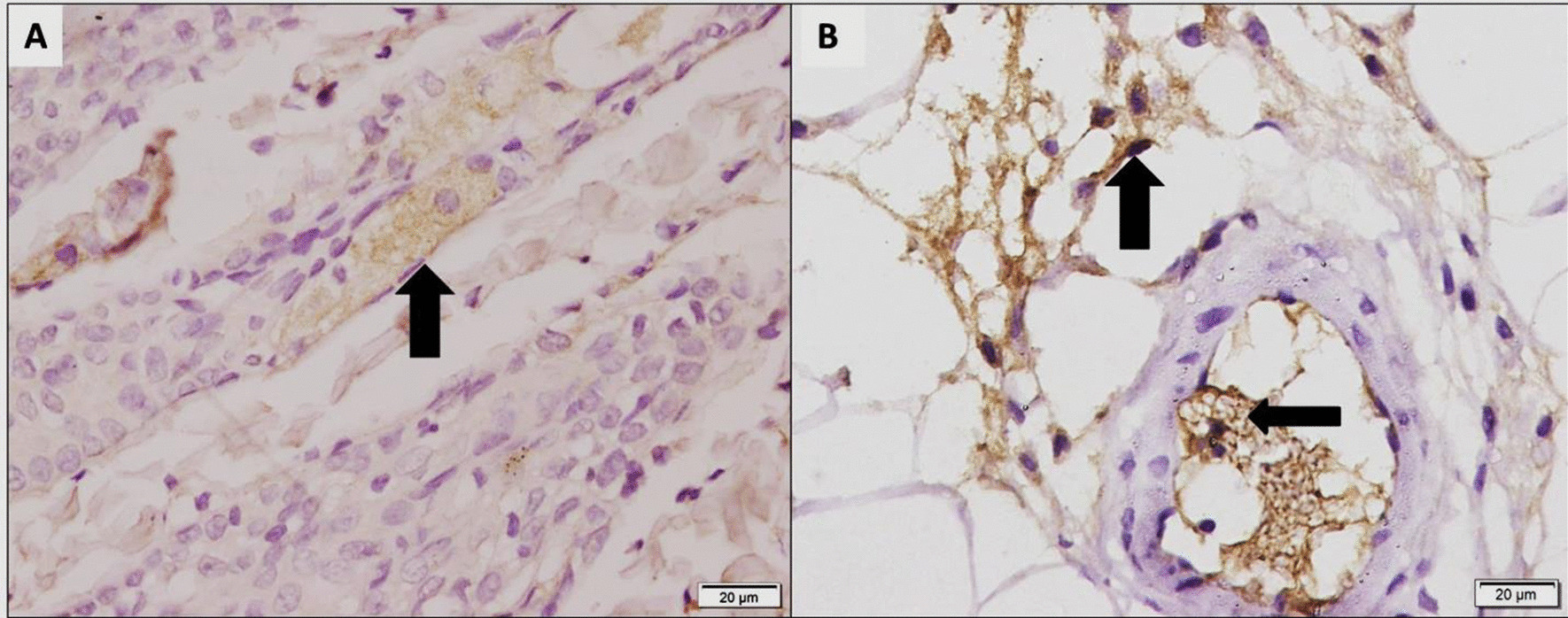
Photomicrographs stained with human anti-globulin (HAG) showing in (**a**): group II (subcutaneous group) +ve immunostaining cells (arrow) detected at the shaft of hair follicle and in (**b**): group III (intraperitoneal group) +ve immunostaining cells detected at the scaffold and blood vessels (arrows) (× 400)

Examination of skin sections from Group I (control group) showed in H&E-stained sections normal epidermal and dermal layer with normal hair follicles and thin keratin layer while showed in immunostained sections −ve skin reaction. Examinations of skin sections from Group II (subcutaneous group) showed in H&E-stained sections hepatocyte-like cells at the bases of the hair follicles. In sections, stained with AFP showed +ve immunostaining cells detected at the roots of hair follicles. While in sections stained with CK19 showed +ve immunostaining cells detected at the shafts of hair follicles (Fig. 5). Examinations of intraperitoneal sections from Group I (control group) showed in H&E-stained sections normal intraperitoneal tissue with some adipose cells and some muscle fibers within loose CT layer while in immunostained sections showed −ve intraperitoneal reactions. Examinations of H&E-stained sections from Group III (intraperitoneal group) showed hepatocytes-like cells at the scaffold with the dilated blood vessel. In both sections stained with AFP and with CK19 showed +ve immunostaining cells detected at the scaffold (Fig. 6). Examinations of sections stained with HAG showed in Group II (subcutaneous group) +ve immunostained cells detected at the shaft of hair follicle and Group III (intraperitoneal group) showed +ve cells detected at the scaffold and blood vessels (Fig. 7).

### Morphometric results

- On day 14, culture tissue showed a significant decrease in the mean area% of AFP immune positive cells and a significant increase in the mean area% of CK19 immuno +ve cells as compared to day 7 (Table 1).
- Mean area% of AFP +ve cells, area% of CK19 and area% of HAG +ve cells immune-expression in control and experimental groups showed statistically significant increased compared to the group I. Group II showed a significant decrease in the mean area% of AFP and a significant increase in area% of CK19 and in area% of HAG +ve cells as compared to group III (Table 2).

**Table 1:** Area% of AFP and CK19 immunopositive cells in the culture tissue on day 0, 7 and 14

Day	0	7	14
Area% of AFP	–	68.33 ± 0.24	27.62 ± 0.55*
Area% of CK19	–	20.21 ± 0.13	50.09 ± 0.42^#^

Mean ± SD can be placed under the table. * Significant decrease compared to day 7 (*P* < 0.001), # significant increased compared to day 7 (*P* < 0.001)

**Table 2:** Mean area% of AFP +ve cells, area% of CK19andarea% of human antiglobulin +ve cells immunoexpression in control and experimental groups ± SD

Group	Area % of AFP +ve cells	Area % of CK19 +ve cells	Area % of human antiglobulin +ve cells
Group I (skin) (peritoneal)	–	–	–
Group II (subcutaneous)	45.1 ± 1.49*	85.60 ± 4.12*	62.54 ± 5.37*
Group III (intraperitoneal)	79.5 ± 4.63*^#^	43.16 ± 6.01*^^^	30.13 ± 3.02*^^^

^*^Significant increased compared to the group I (*P* < 0.001). # Significant increased compared to the group II (*P* < 0.001). ^ Significant decreased compared to the group II (*P* < 0.0)

### Enhanced production of human factor VIII with implantation of bioscaffolds

Therapeutic potential of implanted bioscaffolds documented production of human factor VIII in group II and III. However, serum level of HF VIII was significantly increased in rats subjected to subcutaneous compared to animals received bioscaffold by intraperitoneal implantation (Fig. 2).

## Discussion

The biologically developed scaffolds from animal organs by the process of decellularization can be used to generate a transplantable fully functioning organ [17]. In the present work, we used decellularized pig liver as a bioscaffold for transplantation of hepatocyte-like cells (HLCs) differentiated from human bone marrow mesenchymal stem cells (MSCs). The concept of using decellularized scaffolds was explained by Uygun et al. [17] who stated, these scaffolds can provide a 3D environment helping cell attachment, proliferation, and promotes its function. In our work, the transplanted HLCs were capable of achieving their function in vivo by secreting human factor VIII that was detected in serum samples of the experimental rats.

It was reported that human MSCs from bone marrow, umbilical cord blood, adipose tissues, and fetal bone marrow can differentiate to HLCs [18]. The benefits of MSCs were clarified by Croce et al. [19] who mentioned that MSCs are known by simple isolation, high proliferation abilities and can differentiate into HLCs under proper culture conditions in vitro.

Hepatocyte-like cells derived from bone marrow MSCs have the potential to overcome the limitations of primary human hepatocytes for clinical application in treating liver diseases [20] such as the quality and variability of their metabolic/functional activity. Even more, hepatocytes can’t be easily maintained in culture for extended time [21].

Growth factors can selectively alter cell function and promote MSC proliferation, migration, and differentiation. Accordingly, researchers have tested different protocols to differentiate these cells into other cell types such as hepatocytes. Different growth factors and cytokines are used for sequential differentiation such as FGF, HGF, oncostatin M, and dexamethasone [10].

In the present study, HBMSCs were isolated, cultured and then differentiated into HLCs. On day 14 of the culture, the differentiated cells showed a significant increase in the level of AFP and albumin in the culture media as compared to the control (undifferentiated cells).These results were in accordance to previous Study which confirmed hepatic differentiation by AFP and albumin level [22].

During differentiation, the morphology of HBMSCs changed and the cells expressed hepatocyte-specific antigens as AFP (immature marker) which increased on day 7 and decreased gradually on day 14, on contrary CK19 (mature marker) decreased on day 7 and increased gradually on day 14.These findings agreed with previous work reported that, AFP is an immature marker and albumin synthesis is a mature marker for hepatocytes [23]. Improvement of liver cyto-architecture is indicted by increasing the expression of albumin and reduced the level of AFP [24]. This is consistent with the fact that AFP is expressed in endoderm and in fetal hepatocytes and that after birth the AFP gene is selectively silenced [25]. CK19 is a marker for hepatic oval cell which is a bipotent progenitor cell that can differentiate into both mature hepatocytes and bile duct epithelial cells [26].

In this study, the clusters of HLCs in the decellularized liver scaffold were implanted subcutaneously (group II) and intraperitoneally (group III). The subcutaneous implantation of the decellularized scaffold presented good histocompatibility [27]. In subcutaneous implantation, there are a few concerns regarding implantation such as degradation and inflammation [28].

Implantation of biomaterials stimulates the host immune reaction and enhances recruitment of macrophages that could be attracted by the local bulk of pro-inflammatory cytokines and growth factors allowing monocyte infiltration. The activated immune system release of pro-fibrogenic factors that recruit fibroblasts leading to the final outcome of fibrotic deposition and compromising functions of the implant [29]. In addition, other studies demonstrated stimulation of inflammatory and immune responses after implantation of acellular scaffolds in animal models. The decellularization approaches induce elimination of antigenic parts of the tissues such as the nucleus and other structural elements. However, the extracellular matrix component of the scaffold could stimulate the host immune response, [30, 31] causing accumulation of myofibroblasts leading eventually to fibrosis [32].

Moreover, the peritoneal membranes are covered by mesothelial cells that have the ability of mesothelial-to-mesenchymal transition, which is a type of extracellular matrix. The latter contributes to development of peritoneal fibrosis upon chronic irritation [32, 33]. Thus, it may weaken therapeutic efficacy of intraperitoneal implant.

## Conclusion

Decellularization strategy for synthesis of bioscaffold seeded with hepatocyte-like cells has the potential to present an alternative for recombinant factor VIII replacement to treat hemophilia patient. Increased mean values of serum FVIII in groups subjected to subcutaneous implantation make subcutaneous more preferable than intraperitoneal one.

## Data Availability

The data that support the findings of this study are available from the corresponding author on reasonable request.

## Abbreviations

ECM: Extracellular matrix
FVIII: Factor VIII
MSCs: Mesenchymal stem cells
HGF: Hepatocyte growth factor
FGF: Fibroblast growth factor
AFP: Alpha-fetoprotein
CK19: Cytokeratin 19
HAG: Human anti-globulin
HLCs: Hepatocyte-like cells
HBMSCs: Human bone marrow mesenchymal stem cells
